# Upper Urinary Tract Clot Obstruction After Delayed Hemorrhage Following Partial Nephrectomy: A Possible Association With Tranexamic Acid

**DOI:** 10.7759/cureus.106136

**Published:** 2026-03-30

**Authors:** Ioannis Manolitsis, Sotirios Gatsos, Konstantinos Dimitropoulos, Anuj D Dangi, Grigorios Athanasiadis

**Affiliations:** 1 Urology, Aberdeen Royal Infirmary, Aberdeen, GBR

**Keywords:** gross hematuria, pseudoaneurysm, robotic partial nephrectomy, tranexamic acid, upper urinary tract obstruction

## Abstract

We report a case of delayed hemorrhage occurring four weeks after robotic-assisted partial nephrectomy for renal cell carcinoma. The patient, a 59-year-old lady, presented with gross hematuria and hemodynamic instability. Computed tomography (CT) angiography demonstrated a pseudoaneurysm arising from the resection site. As part of the major hemorrhage protocol, the patient received blood products and tranexamic acid (TXA) for hemorrhage control. Definitive hemostasis was achieved through the selective angioembolization of the pseudoaneurysm.

Following embolization, the patient developed upper urinary tract obstruction secondary to extensive intraluminal clot formation. Endoscopic evaluation revealed a significant clot burden within the bladder and ureter, necessitating clot evacuation and ureteric stent placement to restore urinary drainage. The patient recovered fully, with stable renal function and no recurrence of bleeding following stent removal.

This case highlights a potential association between tranexamic acid use and extensive intraluminal clot formation in the setting of upper urinary tract bleeding, particularly when bleeding communicates directly with the collecting system.

## Introduction

Tranexamic acid (TXA), a synthetic lysine analogue antifibrinolytic, was initially patented in 1957. The mechanism of action involves the competitive inhibition of plasminogen activation by adhering to a lysine binding site and blocking conversion to plasmin. Therefore, TXA reduces fibrinolysis, essentially the enzymatic breakdown of fibrin within blood clots, and stabilizes coagulation while decreasing bleeding [1].

TXA can be delivered through enteral, intravenous, or topical methods. It was first used in clinical settings to address bleeding diseases, including hemophilia, and minimize blood loss during oral surgery. It has been used to avoid traumatic hemorrhage, treat gynecologic bleeding issues and postpartum hemorrhage, and manage gastrointestinal bleeding, without increasing the risk for thromboembolic events [1-3].

While the clinical benefit of TXA has been demonstrated in the context of percutaneous nephrolithotomy (PCNL), there is a paucity of evidence documenting the use of TXA and the related outcomes and adverse effects in patients undergoing other types of renal surgery [4]. This case highlights the potential complications of TXA use in the setting of delayed post-partial nephrectomy hemorrhage.

## Case presentation

**Figure 1:**
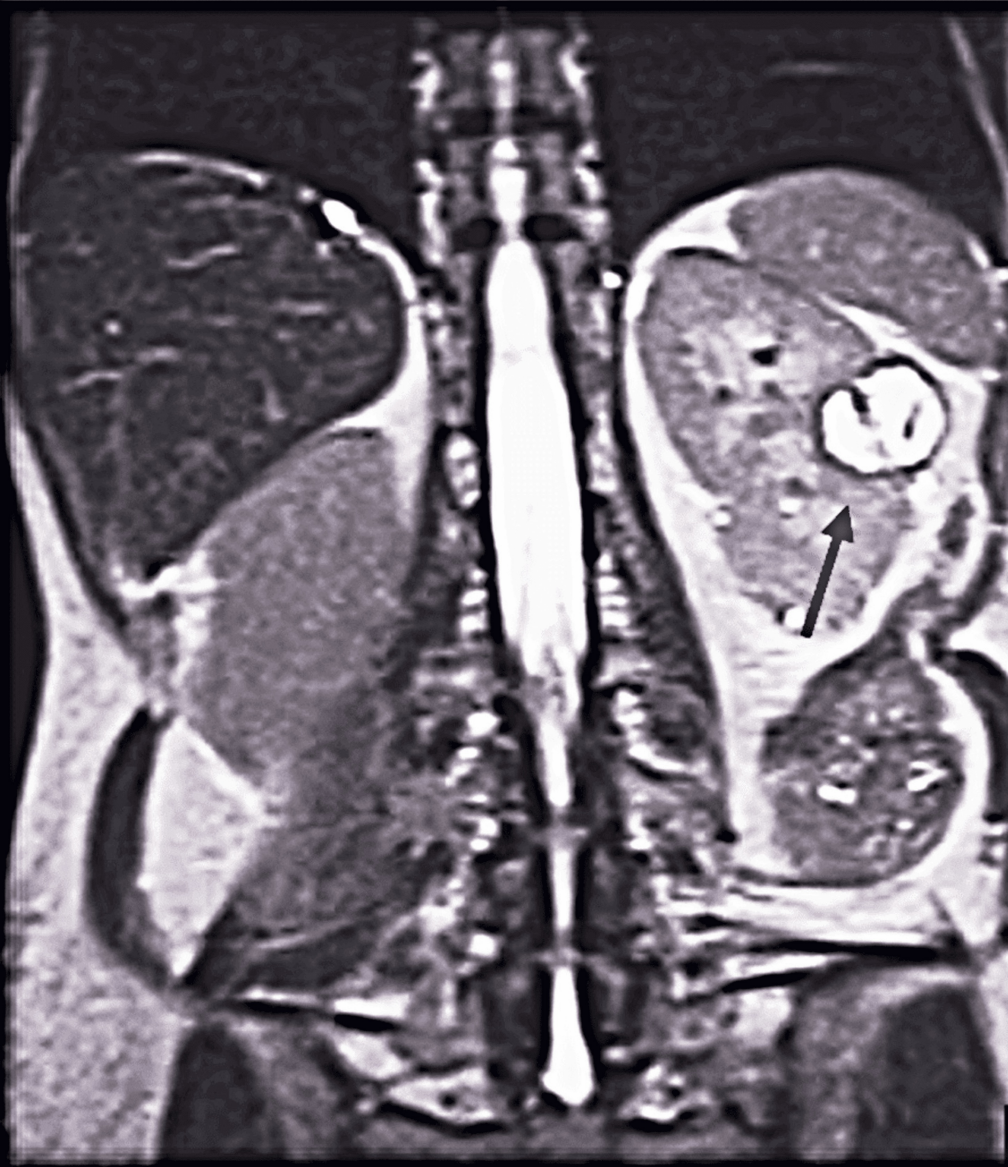
Renal MRI scan showing a 3 cm left renal mass

A 59-year-old female patient was admitted for an elective robotic retroperitoneal left partial nephrectomy. She had a history of depression, hypertension, hypothyroidism, epilepsy, and smoking and an American Society of Anesthesiologists (ASA) score of 2. The patient was initially placed on an active surveillance protocol two years back for a renal mass of 1.8 cm, but after interval increases in size, it was decided that surgery was preferable for the now 3 cm lesion (Figure 1).

Following a successful operation, during which the collecting system was opened and repaired after the tumor excision (warm ischemia time was 16 minutes and estimated blood loss was approximately 25 mL), and an uneventful recovery, she was deemed fit for discharge on postoperative day 1. At discharge, blood tests showed a hemoglobin (Hb) level of 111 g/L, a creatinine of 69 μmol/L, and an estimated glomerular filtration rate (eGFR) of >60 mL/minute/1.73 m^2^.

After a period of four weeks, she presented to the emergency department with frank hematuria and left-sided flank pain. On admission, she reported a reduced urine output. Her hemoglobin level was 90 g/L. Shortly after presentation, she clinically deteriorated with hypotension and tachycardia. Thus, the major hemorrhage protocol was initiated. The resuscitation procedure consisted of administering five units of red blood cells, one unit of fresh frozen plasma (FFP), one unit of platelets, and 1 g of intravenous TXA, followed by 1 g more shortly after, as per protocol.

**Figure 2:**
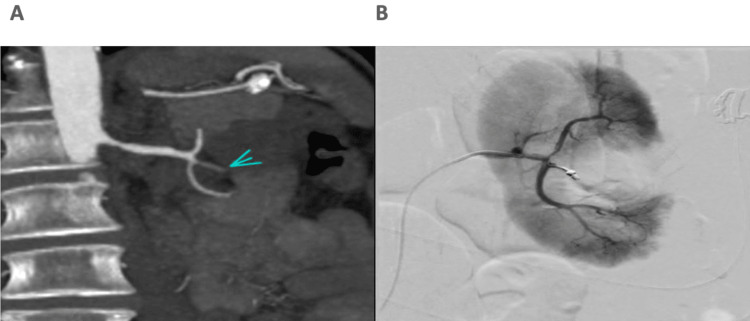
Left renal hematoma with pseudoaneurysm and post-embolization image (A) CT angiography demonstrating the pseudoaneurysm and the hematoma in the left kidney. (B) Digital subtraction angiography of the same area post-embolization CT: computed tomography

The patient underwent a computed tomography (CT) angiography that demonstrated a large pseudoaneurysm (a contained arterial leak caused by blood vessel wall injury), arising from the middle renal artery branch corresponding to the area of the partial nephrectomy. Consequently, a selective embolization of the feeding vessel and the pseudoaneurysm was performed (Figure 2). Within two hours after the procedure, the patient experienced further ongoing hematuria and went into clot retention.

**Figure 3:**
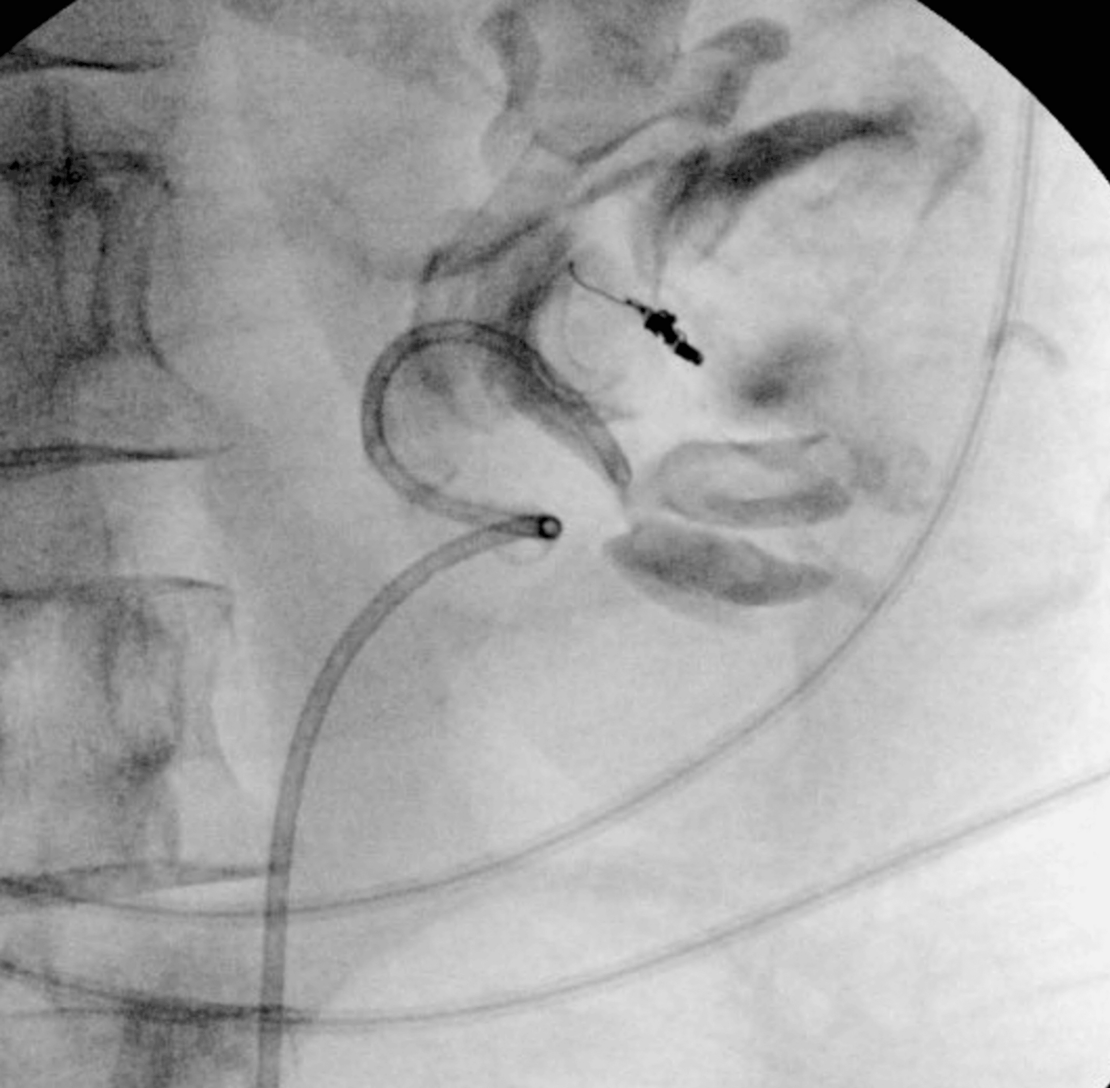
X-ray showing the left ureteric stent in situ

Thereafter, the patient was taken to the theatre for cystoscopy under general anesthesia. The bladder was found to have an extensive clot burden (>300 mL), with clots coming out of the left ureteric orifice. The clots were broken down using a bipolar resectoscope loop and removed using an Ellik evacuator and continuous bladder irrigation. Moreover, a double-J ureteric stent of 6 Fr × 26 cm was inserted in the left collecting system to ensure proper drainage (Figure 3). The patient then had an uncomplicated postoperative course of four days. The blood tests at discharge revealed an Hb level of 107 g/L, an estimated glomerular filtration rate (eGFR) of >60 mL/minute/1.73 m^2^, and a coagulation screen that was normal: the international normalized ratio (INR) was 1.1, prothrombin time was 11.9 seconds, and the activated partial thromboplastin time was 23.3 seconds.

The histopathology report from the partial nephrectomy specimen revealed a pT1a clear cell renal carcinoma with clear margins. She had the ureteric stent removed four weeks after the insertion, and further blood tests after the stent removal showed a normal renal function with an Hb of 127g/L.

The patient is currently on a regular follow-up protocol with CT scans, having shown no recurrence nearly three years after the events. Furthermore, renal function remains normal with no compromise in the radiological appearance of the affected kidney.

## Discussion

Delayed hemorrhage following partial nephrectomy occurs in 1%-2% of cases, usually presenting 4-14 days postoperatively. The predominant causes include vascular injuries, renal artery pseudoaneurysm (RAP), arteriovenous fistula (AVF), and arterial extravasation. Factors related to surgery that elevate the risk include an extensive tumor-parenchyma contact area, increased blood loss, prolonged operating duration, open surgical technique, left-sided procedures, and multifocal tumors [5,6].

There is moderate evidence to support the use of tranexamic acid in the management of hematuria, particularly in surgical and life-threatening situations, but further high-quality trials are needed to determine its efficacy and safety for routine usage in this clinical context [7].

Randomized studies and systematic reviews have shown that TXA lowers blood loss and transfusion requirements in surgical and trauma patients, with no substantial increase in thrombotic events or renal impairment identified. Moreover, a pilot randomized trial found that intravenous TXA significantly reduced the volume of bladder irrigation required to clear urine in patients with painless hematuria but had no effect on hemoglobin drop or transfusion rates. A comprehensive evaluation of TXA in hematuria indicated no increased risk of renal failure due to urinary tract clots, but it did emphasize the lack of robust evidence and the need for further research [7-9]. A shortcut review concluded that TXA may have a role in life-threatening hematuria, especially in patients with autosomal dominant polycystic kidney disease, but the evidence base is limited [10].

The incidence of collecting system complications such as clot retention or ureteric obstruction following tranexamic acid administration in patients with pre-existing renal or urological disorders is very rare but likely underreported, with most evidence limited to case reports and small series. In a case series and literature review, clot retention causing acute obstructive uropathy was described in a kidney transplant patient with chronic renal disease who received unadjusted oral tranexamic acid; the complication improved after discontinuing the medicine and undergoing urological intervention. No incidences of collecting system perforation were found, with thrombotic complications being uncommon in this population [11]. On the contrary, there is a case report presenting the perforation of the upper urinary tract due to clot obstruction caused by TXA [12]. In our case, the sequential association between TXA administration, the presence of a pseudoaneurysm communicating with the collecting system, and subsequent clot obstruction suggests a plausible mechanistic relationship.

Studies evaluating the use of tranexamic acid in patients with hematuria or undergoing PCNL have not managed to demonstrate an increased risk of renal failure or collecting system obstruction compared to controls. However, these studies often lack the detailed reporting of urological complications and exclude patients with advanced kidney disease. Most randomized trials in perioperative settings exclude patients with severe renal impairment (e.g., creatinine clearance <30 mL/minute), and only a few modify dosage for kidney function, limiting the generalizability of safety results to high-risk populations. Tranexamic acid is predominantly excreted in the urine, and accumulation may occur in patients with impaired renal function, potentially increasing the risk of excessive clot stabilization. Therefore, dose adjustment according to renal function is important. A suggested dosing adjustment according to eGFR is provided to improve clinical applicability (Table 1) [13].

**Table 1:** TXA dosing table (renal impairment) Approximate eGFR-based dosing (extrapolated from serum creatinine cutoffs) eGFR, estimated glomerular filtration rate; IV, intravenous; CKD, chronic kidney disease; TID, three times a day; BID, twice a day; TXA, tranexamic acid; q48h, every 48 hours; q24h, every 24 hours

Approximate eGFR (mL/minute/1.73m^2^)	Serum creatinine (mg/dL)	Oral dosing	IV dosing
≥60 (CKD 1-2)	≤1.4	1,300 mg TID	10 mg/kg TID
30-59 (CKD 3)	1.4-2.8	1,300 mg BID	10 mg/kg BID
15-29 (CKD 4)	2.8-5.7	1,300 mg daily	10 mg/kg daily
15 (CKD 5)	>5.7	650 mg daily	10 mg/kg q48h or 5 mg/kg q24h

Risk factors for renal collecting system adverse effects include pre-existing chronic kidney disease, kidney transplantation, unadjusted tranexamic acid dose in renal impairment, and the presence of severe hematuria or a solitary kidney. The available data do not support a significant increase in the incidence of these complications with appropriate dosing and patient selection, but rare events may be missed due to underreporting and exclusion criteria in clinical trials [14,15].

The British National Formulary (BNF) does not specify any urology-specific indications for the use of TXA; however, hematuria is not among the contraindications. The BNF does advise caution in cases of "massive hematuria" (avoid if the risk of ureteric obstruction exists), but it does not specify what constitutes "massive hematuria" or define "risk of ureteric obstruction." On a similar note, the US Food and Drug Administration only acknowledges severe menstrual bleeding and short-term bleeding management in patients with hemophilia as indications for TXA use, even though various non-urological off-label uses have been recorded in the literature [16,17].

In the present case, TXA was administered as part of a major hemorrhage protocol in the context of hemodynamic instability. Although tranexamic acid may have contributed to clot formation and obstruction in this case, alternative explanations should be considered. Delayed hemorrhage following partial nephrectomy is most commonly caused by pseudoaneurysm or arteriovenous fistula, which can result in significant hematuria and clot formation. In addition, clot obstruction can occur in the absence of antifibrinolytic therapy due to ongoing bleeding and urinary stasis. However, the temporal relationship between TXA administration, the embolization of a pseudoaneurysm communicating with the collecting system, and the subsequent development of an extensive intraluminal clot burden raises the possibility that antifibrinolysis may have exacerbated clotting within the urinary tract. TXA does not initiate clot formation as it impairs fibrinolysis, which, in the presence of active or recently controlled bleeding, may obstruct natural clot breakdown and clearance from the collecting system.

## Conclusions

In conclusion, this case highlights a potential association between TXA administration and upper urinary tract clot obstruction in selected high-risk clinical scenarios. The awareness of this possible complication is important, particularly when bleeding communicates directly with the collecting system. Early recognition and prompt urological intervention, including endoscopic clot evacuation and ureteric stenting, are essential to prevent renal impairment and ensure favorable outcomes.
